# Comparison of SARS-CoV-2 immune responses following vaccination with Comirnaty (Pfizer) and Vaxzevria (AstraZeneca) in healthy individuals with or without prior SARS-CoV-2 infection

**DOI:** 10.3389/fimmu.2025.1612288

**Published:** 2025-07-17

**Authors:** Zdzisława Kondera-Anasz, Emilia Justyna Morawiec, Roksana Duszkiewicz, Filip Hajdrowski, Andrzej Wiczkowski

**Affiliations:** ^1^ Department of Immunology, Faculty of Medicine in Zabrze, Academy of Silesia, Katowice, Poland; ^2^ Department of Microbiology, Faculty of Medicine in Zabrze, Academy of Silesia, Katowice, Poland; ^3^ Laboratory of Molecular Biology and Virology, Gyncentrum, Katowice, Poland

**Keywords:** Comirnaty BNT162b2, Vaxzevria ChAdOx1-S, COVID-19 vaccines, anti-SARS-CoV-2 IgG, interferon-gamma, immune response

## Abstract

**Introduction:**

This study compares the immune responses of healthy individuals, with or without prior SARS-CoV-2 infection, following vaccination with Comirnaty (Pfizer) and Vaxzevria (AstraZeneca).

**Methods:**

A total of 134 volunteers were analyzed: 71 recipients of Comirnaty (36 with prior infection) and 63 recipients of Vaxzevria (33 with prior infection). Immune responses were assessed after the second and third doses by measuring anti-SARS-CoV-2 IgG levels and interferon-gamma (IFN-γ) production using an IGRA assay.

**Results:**

Significant differences were observed in IgG and IFN-γ concentrations between the vaccine groups. Higher IgG and IFN-γ levels were noted in individuals vaccinated with Comirnaty, especially after the third dose, indicating a stronger T-cell-mediated response. Prior infection enhanced immune responses, as previously infected individuals showed elevated IgG and IFN-γ levels. Hematological analysis revealed differences in immune activation patterns between vaccines, including variations in white blood cell counts, neutrophil-to-lymphocyte ratio (NLR), and platelet-to-lymphocyte ratio (PLR).

**Discussion:**

These findings highlight distinct vaccine-induced immune responses depending on vaccine type, prior infection status, and number of doses administered. They contribute to understanding the differential immune memory elicited by mRNA-based and adenoviral vector-based vaccines and emphasize the importance of booster doses in maintaining robust immunity against SARS-CoV-2.

## Introduction

In Poland, the vaccination campaign started in late December 2020. The Polish National Institute of Public Health approved and licensed the use of three vaccines against COVID-19: two mRNA vaccines— BNT162b2 (Comirnaty, Pfizer—BioNTech) and mRNA-1273 (Spikevax, Moderna)—and one adenoviral vector–based vaccine, ChAdOx1-S (Vaxzevria, AstraZeneca).

The Comirnaty and Spikevax vaccines are lipid nanoparticle–encapsulated mRNA vaccines directed against S1 protein, while the Vaxzevria vaccine is an chimpanzee adenovirus vector (ChAdOx1, serotype Y25)–based vaccine. The S protein was chosen as the major product of vaccine action due to its binding with the ACE 2 protein, which allows endocytosis of the virus inside the cell; however, antibodies against S protein epitopes prevent the endocytosis of the virus (1, 2).

For mRNA vaccines, the degradation of lipid nanoparticles containing mRNA in endosomes releases mRNA molecules that initiate S protein production in the cytoplasm of myocytes.

The S protein is encoded by a conserved region of the viral genome, which contributes to the genetic stability of the virus variants (3).

The presence of S protein affects the production of T lymphocytes, antibodies, and memory lymphocytes in both infected and vaccinated people (4, 5).

The Vaxzevria vaccine employs a chimpanzee adenovirus vector (ChAdOx1), derived from serotype Y25, which is replication-deficient and produced in the human embryonic kidney cell line HEK293, to deliver the gene encoding the SARS-CoV-2 spike (S) protein (6, 7). After the vector enters human respiratory tract cells, the viral DNA is transported into the nucleus, where it is transcribed into mRNA. This mRNA is then exported into the cytoplasm and serves as a template for S protein translation. The expressed S protein is subsequently processed and presented on the surface of antigen-presenting cells, triggering both humoral and cellular immune responses.

The vaccines authorized for use in Poland differ not only in their mode of delivering the S protein genetic information but also in their mechanism of cellular entry. The final target was to produce S protein and succeed in causing a full immunological response combined with memory of antibodies and T lymphocyte production (8, 9). Differences were observed in the ways in which the vaccines generated differential immune memory.

We focused on finding answers to two questions. Do different mechanisms of cellular penetration and S protein synthesis influence the nature of the immune response, particularly in terms of antibody production and memory lymphocytes? How does prior infection affect the intensity of the immune response?

## Materials and methods

### Study design

For this study, we compared humoral and cellular immunity using quantitative IgG anti-SARS-CoV-2 antibody and a specific interferon-gamma (IFN-γ) release assay (IGRA) at defined timepoints: one month after the second dose and one month after the third (booster) dose of BNT162b2 (Comirnaty, Pfizer–BioNTech) and ChAdOx1-S (Vaxzevria, Oxford–AstraZeneca) vaccines.

### Patient selection

The study involved 134 volunteers, 93 females and 41 males, aged from 19 to 85 (mean age: 40.2 ± 16.3) years after they had received COVID-19 vaccination. Exclusion criteria included, pregnancy, autoimmune diseases, and cancer.

The participants were divided into two groups depending on the vaccines they received: 71 received two doses of the Comirnaty vaccine and 63 received one dose of Vaxzevria vaccine. The participants who received the Comirnaty and Vaxzevria vaccines were further divided into two subgroups. Among the 71 participants who had received the Comirnaty vaccine, 36 contracted COVID-19, and 35 of these infections occurred within 15 days following the first vaccine dose, suggesting pre-existing or pre-vaccination exposure. Similarly, in the group of 63 people who had received the Vaxzevria vaccine, 33 contracted COVID-19, and 30 infections were confirmed within 15 days after the first injection, which may indicate infection acquired prior to or around the time of vaccination.

All participants were tested approximately 30 ± 3 days after the second (II) and third (III) dose of the vaccine. The participants received a third dose after 7 months after the second vaccination. The participants who contracted COVID-19 were vaccinated after 3 months after the infection.

The volunteers of the study were recruited at the Gyncentrum, Laboratory of Molecular Biology and Virology in Katowice, and the NZOZ Central Laboratory in Bytom. The research was conducted from August 2021 to June 2022. Prior signed informed consent forms were obtained from all participants for inclusion in this study.

### Laboratory tests

For healthy subjects with or without prior SARS-CoV-2 infection, antibodies anti-SARS-CoV-2 (IgG) concentration, IFN-γ release assay (IGRA), peripheral white blood cell (WBC), neutrophil (NEU), lymphocyte (LYM), monocyte (MON), basophil (BAS), eosinophil (EO) count, NLR, and platelet-to-lymphocyte ratio (PLR) were investigated and compared after second and third doses of Comirnaty and Vaxzevria vaccines, respectively.

### Antibodies anti-SARS-CoV-2 (IgG)

The test was performed using anti-SARS-CoV-2 QuantiVac enzyme–linked immunosorbent assay (ELISA) IgG (CA no.: El 2606-9601–10 G) provided by Euroimmun Medizinische Labordiagnostika, Lűebeck, Germany. The diagnostic kit includes microplate strips, each containing 8 detachable wells that are pre-coated with a recombinant S1 segment of the SARS-CoV-2 spike protein. During the initial step, diluted patient samples are added to the wells for incubation. If the sample is positive, specific antibodies—primarily IgG, and potentially IgA or IgM—will bind to the antigen on the well surface. In a subsequent step, an enzyme-conjugated anti-human IgG is introduced to detect the attached antibodies. This enzyme triggers a color change, indicating a positive reaction.

As measured by ELISA, the concentration of antibodies in the serum was noted in terms of binding affinity units per milliliter (BAU/mL) in accordance with the manufacturer’s instructions. Due to the linear correlation of results expressed in relative units (RU/ml) with the First WHO International Standard, the results from the quantitative sample evaluation can be converted into standardized units. According to WHO specifications, when using ligand-binding assays, IU/ml values referring to the detection of neutralizing antibodies or BAU/ml should be applied. The values expressed in IU/ml and BAU/ml are numerically identical. To convert test results and borderline ranges given in RU/ml into BAU/ml, the values were multiplied by a factor of 3.2. According to the manufacturer’s instructions, results are interpreted as negative when they are below 25.6 BAU/ml, borderline when between 25.6 and 35.2 BAU/ml, and positive when equal to or above 35.2 BAU/ml.

### Interferon-gamma release assay

SARS-CoV-2-specific T-cell responses were assessed using whole blood IFN-γ (IGRA). The test was performed using Quan-T-Cell SARS-CoV-2 (ca no.: ET 2606-3003) and the levels of IFN-γ in the plasma were noted using the Quan-T-Cell ELISA (ca no.: EQ 6841-9601), both provided by Euroimmun Medizinische Labordiagnostika, Lűebeck, Germany. In the study protocol, whole blood from the patient was incubated for 24 hours in the presence of the SARS-CoV-2 antigen (recombinant receptor-binding domain [RBD] of the S1 spike protein), enabling activation of antigen-specific T cells and subsequent IFN-γ release. The test includes internal controls: a negative control (Blank) — incubation without antigen to determine basal IFN-γ secretion, and a positive control (Stimulant) — a nonspecific mitogen (e.g., SEB – staphylococcal enterotoxin B), which stimulates all T lymphocytes to verify the functional capacity of the cellular immune response. After incubation, the supernatant is collected and quantitatively analyzed using the EUROIMMUN Anti–IFN-γ ELISA. The antibodies and IFN-γ concentrations were evaluated using an automated VICTOR Nivo Multimode Plate Reader (PerkinElmer, USA) analyzer at 450 nm. According to the manufacturer’s instructions for use (IFU), responses < 100 mIU/ml are typically classified as negative, ≥ 200 mIU/ml as positive, and intermediate values as borderline.

According to the manufacturer’s declaration, despite the high specificity of the applied antigen (recombinant RBD domain of the SARS-CoV-2 S1 protein), cross-reactions with other seasonal coronaviruses (e.g., HCoV-OC43, HCoV-229E) cannot be completely ruled out. This may lead to positive results in individuals who have not been infected with SARS-CoV-2 but have had prior exposure to other coronaviruses sharing conserved epitopes within the RBD region.

### Blood count

Peripheral WBC, neutrophil (NEU), lymphocyte (LYM), monocyte (MON), basophil (BAS), and eosinophil (EO) counts were used to calculate the following indicators: NLR and PLR. NLR is defined by the absolute number of neutrophils divided by the absolute number of lymphocytes, while PLR is the ratio between absolute platelet and lymphocyte counts.

### Statistical analysis

The results were statistically analyzed using the computer program Statistica for Windows 13.36 and Microsoft Excel. The Shapiro–Wilk test was used to verify the distribution of the obtained results, and the data were presented in terms of medians and interquartile range (IQR). *Post-hoc* comparisons were conducted using Dunn’s test with Bonferroni correction after obtaining a significant result in the Kruskal-Wallis test. A probability level of *p* < 0.05 was considered to be statistically significant.

## Results

**Figure 1:**
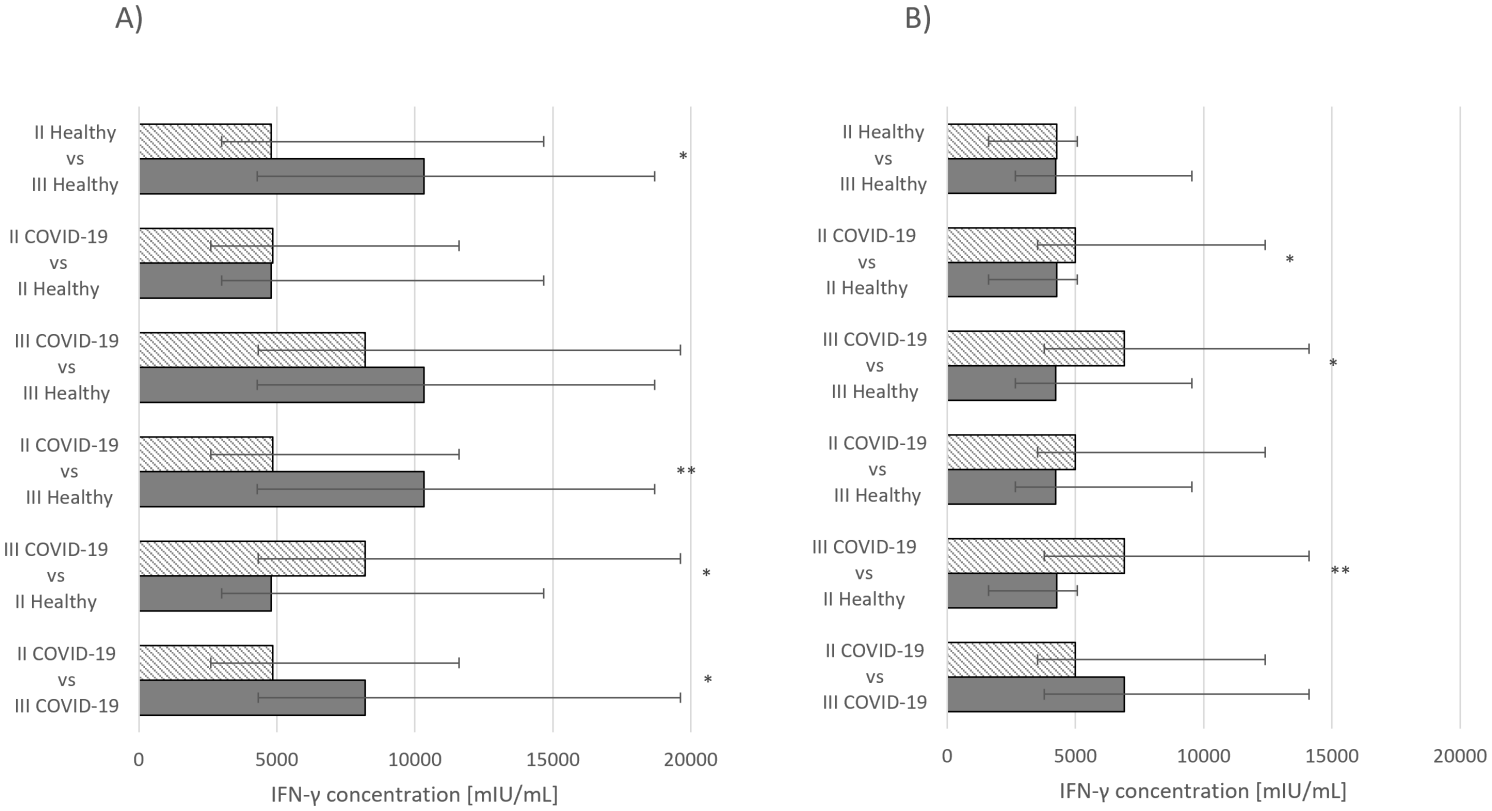
**(A)** IFN-γ concentration median values *Comirnaty vaccine*. **(B)** IFN-γ concentration median values *Vaxzevria vaccine*. *p ≤ 0.05, **p ≤ 0.01.

Differences between statistical significances of IgG concentrations were observed in individuals who had received second and third doses of Comirnaty without prior COVID-19 infection and those who had recovered from the disease and had received second and third doses (*p* ≤ 0.001) (**Figure 1A**). When assessing the same parameter in individuals vaccinated with Vaxzevria, statistically significant differences were noted between healthy individuals who had received second and third doses (*p* ≤ 0.01) and those who had recovered from COVID-19 and received second and third doses (*p* ≤ 0.001) (**Figure 1B**).

**Figure 2:**
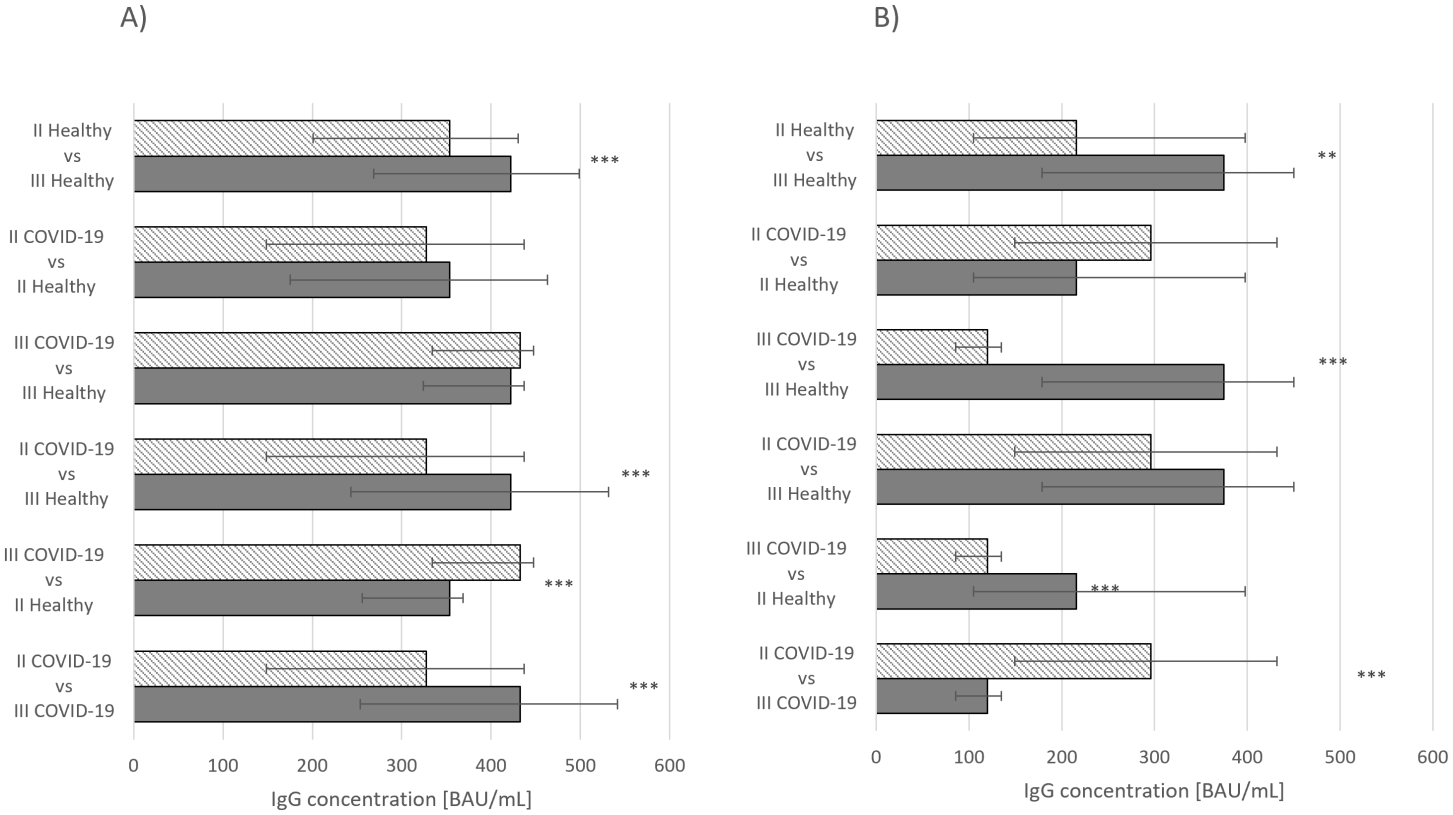
**(A)** IgG concentration median values *Comirnaty vaccine*. **(B)** IgG concentration median values *Vaxzevria vaccine*. ***p ≤ 0.001.

Statistically significant differences in IFN-γ concentrations were also observed. For the Comirnaty vaccine, significance was noted between healthy group that had received second and third doses (*p* ≤ 0.05) and the group that had recovered from COVID-19 and received either second (*p* ≤ 0.05) or third dose (*p* ≤ 0.01) (**Figure 2A**). In the group receiving Vaxzevria, statistically significant differences were observed in individuals who had recovered from COVID-19 and received either second (*p* ≤ 0.05) or third dose (*p* ≤ 0.01) (**Figure 2B**).

**Figure 3:**
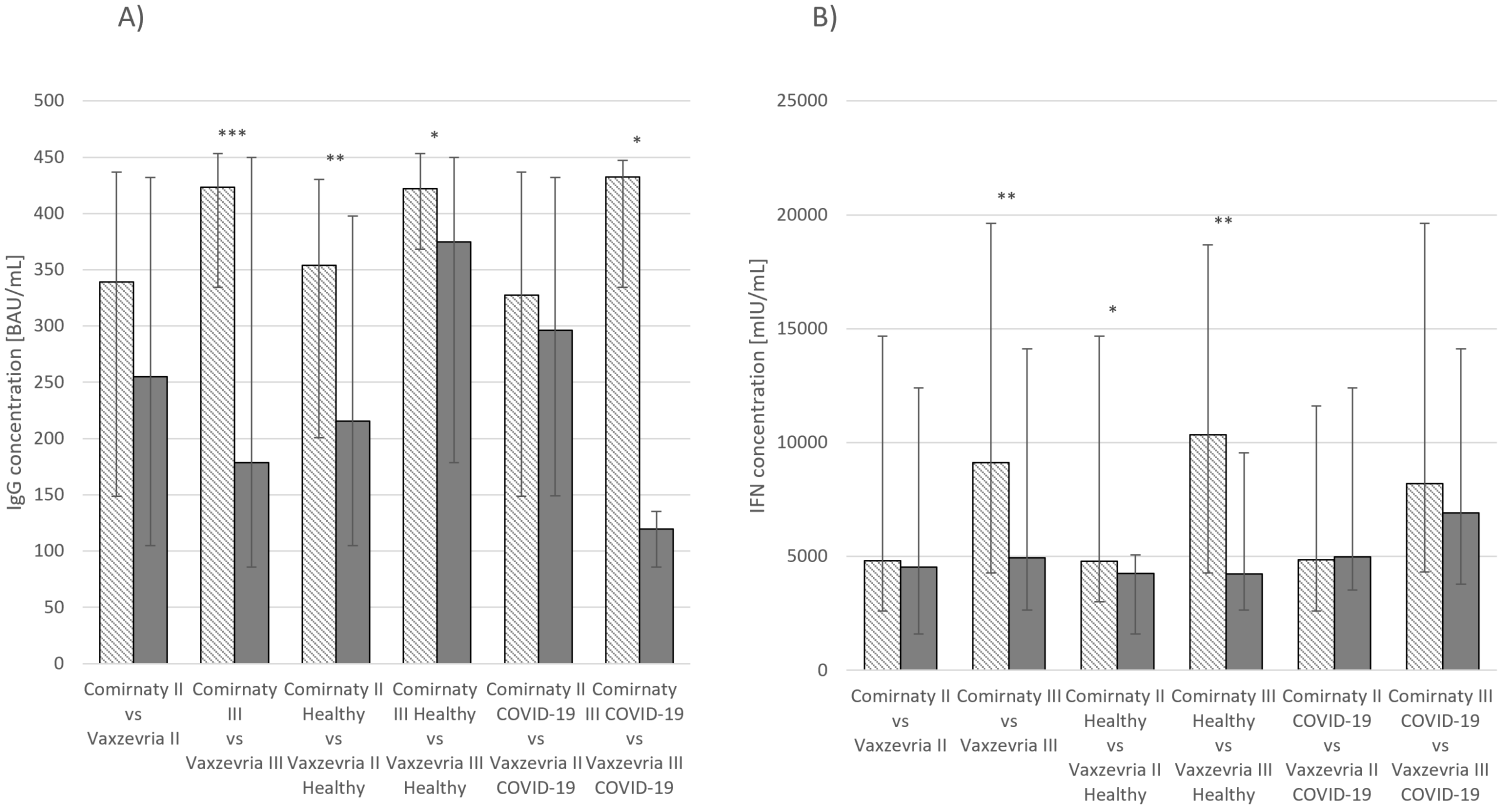
**(A)** IgG concentration median values *Comirnaty* and *Vaxzevria vaccine*. **(B)** IFN-γ concentration median values *Comirnaty* and *Vaxzevria vaccine*. *p ≤ 0.05, **p ≤ 0.01, ***p ≤ 0.001.

IgG and IFN-γ concentrations were also compared between groups receiving the two vaccines. Statistically significant differences in IgG concentrations were observed among healthy individuals and those who had recovered from COVID-19 after receiving the third dose (*p* ≤ 0.05), healthy individuals who had received the second dose (*p* ≤ 0.01), and the overall group of individuals who had received the third dose (*p* ≤ 0.001) (**Figure 3A**). Similarly, for IFN-γ concentrations, significant differences were observed between individuals who had received the third dose of either vaccine (*p* ≤ 0.01) and healthy individuals who had received second (*p* ≤ 0.05) or third (*p* ≤ 0.01) dose (**Figure 3B**).

In most of the studied groups, statistically significant differences were observed in IgG and IFN concentrations (**Table 1**). The following parameters showed statistically significant changes:

- WBC: In healthy individuals (*p* ≤ 0.05) and those who had recovered from COVID-19 (*p* ≤ 0.01), significant differences were observed between groups of individuals who had received Comirnaty and those who had received Vaxzevria as a third dose.
- PLT: Differences were found between healthy individuals (*p* ≤ 0.05) who had received Comirnaty and Vaxzevria as a third dose and those who had recovered from COVID-19 and received the second dose (*p* ≤ 0.05). Overall, significant differences were also observed between the groups who had received second or third doses (*p* ≤ 0.05).
- NEU: Differences were noted between healthy individuals (*p* ≤ 0.05) who had received Comirnaty and those who had received Vaxzevria as second or third dose.
- LYM: Differences were observed between individuals who had received Comirnaty and those who had received Vaxzevria as a third dose and had recovered from COVID-19 (*p* ≤ 0.05) and between the groups that had received second or third doses (*p* ≤ 0.01).
- EO: Significant differences were found between groups that had received the second or third doses (*p* ≤ 0.001).
- BAS: Differences were observed between groups that had received second or third doses (*p* ≤ 0.05).
- NLR: Differences were found in individuals who had recovered from COVID-19 and received either vaccine and all vaccinated individuals (*p* ≤ 0.05). Furthermore, a significant difference was observed between individuals who had received Comirnaty and those who had received Vaxzevria as a second dose in healthy individuals (*p* ≤ 0.05).
- PLR: Differences were also found in healthy individuals who had received either vaccine and those who had recovered from COVID-19. A significant difference was also observed between individuals who had no prior COVID-19 infection (*p* ≤ 0.05) and had received Comirnaty and/or Vaxzevria as a second dose.

**Table 1:** Concentrations of immunoglobulin G (IgG), interferon-gamma (IFN-γ), and other morphological parameters of individual study groups.

Parameters		COM vs. VAX	Healthy vs. COVID-19	II vs. III	COM/healthy vs. VAX/healthy	COM/COVID-19 vs. VAX/COVID-19	COM/II vs. VAX/II	COM/III vs. VAX/III	COM vs. VAX healthy/II	COM vs. VAX healthy/III	COM vs. VAX COVID-19/II	COM vs. VAX COVID-19/III
IgG [BAU/mL]	median (range); [IQR]	380.80 (148.80, 453.10); [320.95, 422.90]	364.20 (105.00, 453.10); [230.70, 408.60]	296.30 (105.00, 436.80); [203.35, 361.80]	389.50 (200.60, 453.10); [344.73, 421.65]	371.20 (148.80, 447.40); [281.60, 429.90]	339.05 (148.80, 436.80); [211.58, 373.65]	423.40 (334.40, 453.10); [392.65, 439.05]	353.75 (200.60, 430.40); [250.73, 386.60]	421.90 (368.30, 453.10); [399.88, 435.55]	327.55 (148.80, 436.80); [193.00, 360.53]	432.30 (334.40, 447.40); [387.20, 444.80]
		230.70 (85.90, 449.90); [134.55, 357.95]	296.30 (85.90, 447.40); [149.10, 380.80]	390.65 (85.90, 453.10); [186.28, 429.68]	255.40 (105.00, 449.90); [203.50, 395.20]	142.10 (85.90, 432.00); [120.28, 288.15]	254.90 (105.00, 432.00); [196.40, 353.70]	178.60 (85.90, 449.90); [120.75, 374.55]	215.70 (105.00, 397.80); [183.40, 303.00]	374.55 (178.60, 449.90); [241.23, 419.68]	296.30 (149.10, 432.00); [234.85, 361.00]	119.80 (85.90, 135.10); [109.50, 133.55]
	*p*-value	<0.0001***	0.0259*	<0.0001***	0.0023**	<0.0001***	0.0924	<0.0001***	0.0084**	0.0143*	0.7897	<0.0001*
IFN-γ [mIU/mL]	median (range); [IQR]	4982.14 (2603.15, 19623.00); [4434.93, 11022.55]	4768.88 (1604.70, 18693.00); [4155.05, 8125.90]	4753.45 (1604.70, 14667.40); [4041.89, 5482.20]	4898.69 (3003.25, 18693.00); [4501.54, 12539.78]	5206.20 (2603.15, 19623.00); [4331.45, 9763.63]	4805.36 (2603.15, 14667.40); [4272.10, 7751.78]	9116.90 (4284.36, 19623.00); [4704.68, 16815.55]	4788.50 (3003.25, 14667.40); [4425.85, 5874.63]	10336.80 (4284.36, 18693.00); [4743.35, 17139.05]	4846.93 (2603.15, 11606.80); [4154.69, 7797.85]	8200.30 (4326.00, 19623.00); [4849.93, 15221.45]
		4727.81 (1604.70, 14110.44); [3856.21, 7160.22]	5206.20 (2603.15, 19623.00); [4336.90, 9721.10]	6610.69 (2651.20, 19623.00); [4388.66, 11178.69]	4260.10 (1604.70, 9541.62); [3400.00, 4912.19]	5362.95 (3521.46, 14110.44); [4372.26, 9423.87]	4530.74 (1604.70, 12408.56); [3559.46, 5007.81]	4936.90 (2651.20, 14110.44); [3912.39, 8515.200]	4263.45 (1604.70, 5074.44); [3329.92, 4768.88]	4238.15 (2651.20, 9541.62); [3765.54, 6588.69]	4985.60 (3521.46, 12408.56); [4153.40, 7856.88]	6909.82 (3779.15, 14110.44); [4544.67, 10105.15]
	*p*-value	0.0017*	0.0621	<0.0001***	0.0002*	0.6833	0.0698	0.0017**	0.0161*	0.0020**	0.8415	0.2717
WBC × 10^9^/L	median (range); [IQR]	6.37 (3.44, 10.60); [5.41, 7.16]	6.35 (3.44, 9.72); [5.60, 7.45]	6.24 (3.74, 10.63); [5.60, 7.21]	6.26 (3.44, 9.72); [5.31, 6.85]	6.44 (3.74, 10.60); [5.51, 7.33]	6.42 (3.74, 10.60); [5.53, 7.51]	6.18 (3.44, 8.88); [5.30, 7.04]	6.58 (4.58, 9.72); [5.70, 7.75]	5.65 (3.44, 8.38); [5.05, 6.46]	6.02 (3.74, 10.60); [5.28, 6.89]	6.94 (4.68, 8.88); [6.11, 7.49]
		6.12 (4.12, 10.63); [5.46, 7.31]	6.13 (3.74, 10.63); [5.20, 7.13]	6.25 (3.44, 8.94); [5.28, 7.20]	6.46 (4.12, 8.94); [5.71, 7.57]	5.95 (4.62, 10.63); [5.10, 6.67]	6.04 (4.12, 10.63); [5.66, 7.06]	6.27 (4.40, 8.94); [5.29, 7.49]	6.04 (4.12, 7.90); [5.71, 6.50]	7.31 (4.40, 8.94); [6.20, 8.10]	6.44 (4.64, 10.63); [5.12, 7.37]	5.75 (4.62, 7.82); [5.05, 6.25]
	*p*-value	0.7805	0.5085	0.2296	0.3457	0.1611	0.6875	0.8658	0.2531	0.0143*	0.7389	0.0075**
PLT × 10^9^/L	median (range); [IQR]	252.00 (135.00, 415.00); [217.50, 293.00]	250.00 (149.00, 367.00); [221.00, 293.00]	250.50 (135.00, 415.00); [213.00, 290.00]	266.50 (172.00, 367.00); [235.00, 293.50]	247.50 (135.00, 415.00); [209.50, 290.00]	247.50 (135.00, 415.00); [209.50, 290.00]	253.00 (171.00, 392.00); [221.50, 296.50]	273.00 (172.00, 367.00); [242.00, 300.00]	248.00 (188.00, 355.00); [221.75, 289.00]	229.00 (135.00, 415.00); [192.50, 257.50]	265.00 (171.00, 392.00); [218.50, 316.00]
		244.00 (149.00, 364.00); [206.00, 285.50]	250.00 (135.00, 415.00); [205.00, 289.00]	248.00 (153.00, 392.00); [207.50, 291.75]	234.00 (149.00, 364.00); [206.00, 275.00]	252.00 (153.00, 361.00); [207.00, 289.75]	250.50 (149.00, 364.00); [213.00, 289.50]	234.00 (153.00, 361.00); [205.50, 276.00]	238.00 (149.00, 364.00); [206.00, 262.00]	234.00 (161.00, 316.00); [214.25, 282.25]	276.00 (183.00, 349.00); [235.50, 305.00]	223.00 (153.00, 361.00); [196.50, 276.00]
	*p*-value	0.3585	0.6243	0.0440*	0.0652	0.6546	0.9864	0.1857	0.0443*	0.6511	0.0455*	0.1647
NEU × 10^9^/L	median (range); [IQR]	3.70 (1.29, 6.72); [3.02, 4.19]	3.75 (1.29, 6.72); [3.00, 4.48]	3.80 (1.78, 7.52); [2.97, 4.20]	3.73 (1.29, 6.72); [3.10, 4.19]	3.85 (1.78, 6.72); [3.05, 4.68]	3.85 (1.78, 6.72); [3.05, 4.68]	3.50 (1.29, 5.29); [3.00, 3.97]	4.10 (2.40, 6.72); [3.56, 4.90]	3.11 (1.29, 5.29); [2.87, 3.53]	3.15 (1.78, 6.09); [2.61, 3.94]	3.78 (1.99, 4.88); [3.50, 4.25]
		3.75 (1.81, 7.52); [3.01, 4.60]	3.71 (1.78, 7.52); [3.04, 4.26]	3.62 (1.29, 6.72); [3.05, 4.38]	3.75 (1.81, 6.72); [2.95, 4.56]	3.76 (2.33, 7.52); [3.16, 4.55]	3.77 (1.81, 7.52); [2.96, 4.10]	3.75 (1.84, 6.72); [3.05, 4.88]	3.15 (1.81, 5.32); [2.88, 3.88]	4.15 (1.84, 6.72); [3.00, 5.44]	3.84 (2.35, 7.52); [3.51, 4.45]	3.57 (2.33, 5.12); [3.08, 4.45]
	*p*-value	0.5311	0.7452	0.3138	0.9425	0.3780	0.5557	0.1132	0.0189*	0.0479*	0.1518	0.7875
LYM × 10^9^/L	median (range); [IQR]	2.05 (0.95, 3.60); [1.71, 2.28]	1.98 (0.95, 3.13); [1.63, 2.25]	2.05 (0.95, 3.48); [1.79, 2.28]	1.88 (0.95, 3.13); [1.51, 2.16]	2.01 (0.95, 3.48); [1.76, 2.27]	2.01 (0.95, 3.48); [1.76, 2.27]	2.07 (1.21, 3.60); [1.69, 2.35]	1.85 (0.95, 3.13); [1.49, 2.07]	2.01 (1.21, 2.78); [1.56, 2.17]	2.14 (1.24, 3.48); [1.88, 2.32]	2.25 (1.68, 3.60); [2.04, 2.49]
		2.01 (0.98, 2.93); [1.75, 2.30]	2.12 (1.07, 3.60); [1.78, 2.31]	2.02 (0.98, 3.60); [1.69, 2.33]	2.02 (0.98, 2.93); [1.75, 2.36]	1.98 (1.07, 2.85); [1.74, 2.20]	2.14 (1.29, 2.83); [1.81, 2.29]	3.75 (1.84, 6.72); [3.05, 4.88]	2.13 (1.29, 2.83); [1.85, 2.36]	1.90 (0.98, 2.93); [1.74, 2.45]	2.14 (1.56, 2.43); [1.79, 2.22]	1.90 (1.07, 2.85); [1.56, 2.11]
	*p*-value	0.8954	0.1296	0.0024**	0.0974	0.0515	0.4178	0.3384	0.1063	0.4070	0.5375	0.0344*
MON × 10^9^/L	median (range); [IQR]	0.52 (0.21, 0.88); [0.42, 0.62]	0.50 (0.21, 0.85); [0.43, 0.62]	0.52 (0.24, 0.88); [0.42, 0.59]	0.49 (0.21, 0.77); [0.41, 0.61]	0.49 (0.32, 0.88); [0.41, 0.59]	0.49 (0.32, 0.88); [0.41, 0.59]	0.53 (0.21, 0.72); [0.44, 0.62]	0.48 (0.32, 0.77); [0.39, 0.59]	0.52 (0.21, 0.67); [0.43, 0.62]	0.54 (0.32, 0.88); [0.42, 0.59]	0.54 (0.32, 0.72); [0.51, 0.62]
		0.52 (0.24, 0.85); [0.47, 0.61]	0.54 (0.29, 0.88); [0.44, 0.60]	0.52 (0.21, 0.85); [0.46, 0.62]	0.51 (0.24, 0.85); [0.47, 0.62]	0.53 (0.29, 0.82); [0.48, 0.59]	0.53 (0.24, 0.82); [0.48, 0.59]	0.50 (0.29, 0.85); [0.47, 0.62]	0.51 (0.24, 0.76); [0.44, 0.58]	0.52 (0.46, 0.85); [0.48, 0.63]	0.57 (0.41, 0.82); [0.51, 0.61]	0.50 (0.29, 0.76); [0.43, 0.55]
	*p*-value	0.4486	0.2873	0.1263	0.2691	0.9948	0.2717	0.7514	0.6044	0.4070	0.2433	0.1776
EO × 10^9^/L	median (range); [IQR]	0.14 (0.00, 0.57); [0.09, 0.21]	0.15 (0.01, 0.68); [0.09, 0.21]	0.11 (0.01, 0.36); [0.07, 0.18]	0.12 (0.01, 0.36); [0.09, 0.20]	0.12 (0.01, 0.36); [0.07, 0.18]	0.12 (0.01, 0.36); [0.07, 0.18]	0.16 (0.00, 0.57); [0.11, 0.23]	0.10 (0.01, 0.36); [0.06, 0.14]	0.16 (0.04, 0.33); [0.12, 0.22]	0.16 (0.03, 0.27); [0.09, 0.20]	0.16 (0.00, 0.57); [0.10, 0.23]
		0.14 (0.01, 0.68); [0.09, 0.21]	0.14 (0.00, 0.57); [0.09, 0.20]	0.16 (0.00, 0.68); [0.11, 0.23]	0.17 (0.01, 0.68); [0.10, 0.24]	0.12 (0.04, 0.57); [0.08, 0.18]	0.10 (0.01, 0.34); [0.07, 0.18]	0.15 (0.01, 0.68); [0.12, 0.24]	0.51 (0.24, 0.76); [0.44, 0.58]	0.18 (0.01, 0.68); [0.15, 0.26]	0.10 (0.04, 0.30); [0.08, 0.14]	0.12 (0.08, 0.57); [0.10, 0.21]
	*p*-value	0.8707	0.6772	0.0001***	0.2343	0.3401	0.7900	0.8603	0.5729	0.3758	0.2641	0.7244
BAS × 10^9^/L	median (range); [IQR]	0.03 (0.01, 0.09); [0.02, 0.05]	0.03 (0.01, 0.09); [0.02, 0.04]	0.03 (0.01, 0.09); [0.02, 0.04]	0.03 (0.01, 0.09); [0.02, 0.04]	0.03 (0.01, 0.09); [0.02, 0.04]	0.03 (0.01, 0.09); (0.02, 0.04]	0.04 (0.01, 0.09); [0.02, 0.05]	0.04 (0.01, 0.09); [0.03, 0.04]	0.03 (0.01, 0.09); [0.02, 0.04]	0.02 (0.01, 0.08); [0.02, 0.03]	0.04 (0.01, 0.07); [0.03, 0.06]
		0.03 (0.01, 0.07); [0.02, 0.04]	0.03 (0.01, 0.08); [0.02, 0.04]	0.03 (0.01, 0.09); [0.02, 0.05]	0.03 (0.01, 0.06); [0.02, 0.04]	0.03 (0.01, 0.07); [0.02, 0.04]	0.03 (0.01, 0.06); [0.02, 0.04]	0.03 (0.02, 0.07); [0.02, 0.04]	0.03 (0.01, 0.06); [0.02, 0.04]	0.04 (0.02, 0.06); [0.03, 0.05]	0.03 (0.01, 0.05); [0.03, 0.05]	0.03 (0.02, 0.07); [0.02, 0.04]
	*p*-value	0.8410	0.2372	0.0225*	0.6480	0.8179	0.7769	0.4429	0.1702	0.4975	0.1252	0.0890
NLR	median (range); [IQR]	1.84 (0.76, 4.71); [1.42, 2.21]	1.95 (0.76, 4.71); [1.36, 2.59]	1.82 (0.86, 4.71); [1.38, 2.38]	2.01 (0.76, 4.71); [1.67, 2.44]	1.92 (0.86, 4.71); [1.56, 2.39]	1.92 (0.86, 4.71); [1.56, 2.39]	1.69 (0.76, 2.76); [1.27, 2.05]	2.12 (1.32, 4.71); [1.87, 2.64]	1.76 (0.76, 2.76); [1.39, 2.05]	1.63 (0.86, 2.62); [1.34, 2.02]	1.51 (0.96, 2.43); [1.27, 2.02]
		1.91 (0.78, 4.38); [1.37, 2.39]	1.79 (0.86, 3.82); [1.46, 2.21]	1.87 (0.76, 4.38); [1.39, 2.35]	1.79 (0.78, 4.38); [1.31, 2.65]	1.96 (1.11, 3.82); [1.66, 2.36]	1.77 (1.06, 3.51); [1.34, 2.37]	2.04 (0.78, 4.38); [1.65, 2.62]	1.38 (1.06, 2.97); [1.28, 2.05]	2.11 (0.78, 4.38); [1.56, 2.98]	1.91 (1.11, 3.51); [1.56, 2.38]	1.98 (1.18, 3.82); [1.82, 2.28]
	*p*-value	0.3481	0.2408	0.3658	0.4279	0.0172*	0.3706	0.0238*	0.0114*	0.1632	0.1518	0.0712
PLR	median (range); [IQR]	119.89 (67.52, 308.42); [102.49, 152.99]	127.96 (62.08, 308.42); [105.40, 160.09]	117.11 (62.08, 308.42); [97.63, 159.68]	144.08 (90.20, 308.42); [118.44, 167.08]	123.89 (77.63, 308.42); [100.26, 160.09]	123.89 (77.63, 308.42); [100.26, 160.09]	119.75 (67.52, 194.05); [107.16, 149.93]	148.55 (90.20, 308.42); [121.71, 173.38]	137.56 (101.83, 182.64); [118.44, 152.32]	103.61 (77.63, 187.78); [95.69, 122.58]	116.74 (67.52, 194.05); [99.43, 134.44]
		123.70 (62.08, 223.72); [102.33, 151.96]	117.37 (62.46, 223.72); [101.06, 142.20]	126.59 (62.46, 221.43); [107.44, 150.80]	115.68 (62.08, 223.31); [95.34, 135.38]	131.06 (62.46, 223.72); [107.30, 159.08]	1.77 (1.06, 3.51); [1.34, 2.37]	128.37 (62.46, 221.43); [107.55, 144.73]	107.56 (62.08, 223.31); [93.72, 121.89]	130.46 (68.51, 221.43); [109.04, 135.71]	1.91 (1.11, 3.51); [1.56, 2.38]	1.98 (1.18, 3.82); [1.82, 2.28]
	*p*-value	0.7297	0.1273	0.0728	0.0116*	0.0299*	0.6341	0.9159	0.0355*	0.3089	0.0574	0.2902

*p ≤ 0.05, **p ≤ 0.01, ***p ≤ 0.001 statistical significance.

No statistically significant differences were observed in monocyte concentrations across the studied groups (**Table 1**).

Statistically significant differences were observed in IgG and IFN-γ concentrations in the groups that had received Comirnaty and Vaxzevria (**Table 2**).

**Table 2:** Concentrations of IgG, IFN-γ and other morphological parameters in individual study groups, categorized by vaccine type—(A) Comirnaty or (B) Vaxzevria—and vaccine dose (second [II] or third [III]).

Parameters		Healthy vs. COVID-19	II vs. III	II healthy vs. III healthy	II Healthy vs. II COVID-19	II Healthy vs. III COVID-19	III Healthy vs. II COVID-19	III Healthy vs. III COVID-19	II COVID-19 vs. III COVID-19
A) *Comirnaty* vaccine									
IgG [BAU/mL]	median (range); [IQR]	389.50 (200.60, 453.10); [344.73, 421.65]	339.05 (148.80, 436.80); [211.58, 373.65]	353.75 (200.60, 430.40); [250.73, 386.60]	353.75 (200.60, 430.40); [250.73, 386.60]	353.75 (200.60, 430.40); [250.73, 386.60]	421.90 (368.30, 453.10); [399.88, 435.55]	421.90 (368.30, 453.10); [399.88, 435.55]	327.55 (148.80, 436.80); [193.00, 360.53]
		371.20 (148.80, 447.40); [281.60, 429.90]	423.40 (334.40, 453.10); [392.65, 439.05]	421.90 (368.30, 453.10); [399.88, 435.55]	327.55 (148.80, 436.80); [193.00, 360.53]	432.30 (334.40, 447.40); [387.20, 444.80]	327.55 (148.80, 436.80); [193.00, 360.53]	432.30 (334.40, 447.40); [387.20, 444.80]	432.30 (334.40, 447.40); [387.20, 444.80]
	*p*-value	0.4513	<0.0001***	<0.0001***	0.1595	0.0002***	<0.0001***	0.7220	<0.0001***
IFN-γ [mIU/m]	median (range); [IQR]	4898.69 (3003.25, 18693.00); [4501.54, 12539.78]	4805.36 (2603.15, 14667.40); [4272.10, 7751.78]	4788.50 (3003.25, 14667.40); [4425.85, 5874.63]	4788.50 (3003.25, 14667.40); [4425.85, 5874.63]	4788.50 (3003.25, 14667.40); [4425.85, 5874.63]	10336.80 (4284.36, 18693.00); [4743.35, 17139.05]	10336.80 (4284.36, 18693.00); [4743.35, 17139.05]	4846.93 (2603.15, 11606.80); [4154.69, 7797.85]
		5206.20 (2603.15, 19623.00); [4331.45, 9763.63]	9116.90 (4284.36, 19623.00); [4704.68, 16815.55]	10336.80 (4284.36, 18693.00); [4743.35, 17139.05]	4846.93 (2603.15, 11606.80); [4154.69, 7797.85]	8200.30 (4326.00, 19623.00); [4849.93, 15221.45]	4846.93 (2603.15, 11606.80); [4154.69, 7797.85]	8200.30 (4326.00, 19623.00); [4849.93, 15221.45]	8200.30 (4326.00, 19623.00); [4849.93, 15221.45]
	*p*-value	0.6579	0.0009***	0.0280*	0.7150	0.0329*	0.0090**	0.7820	0.0164*
WBC × 10^9^/L	median (range); [IQR]	6.26 (3.44, 9.72); [5.31, 6.85]	6.42 (3.74, 10.60); [5.53, 7.51]	6.58 (4.58, 9.72); [5.70, 7.75]	6.58 (4.58, 9.72); [5.70, 7.75]	6.58 (4.58, 9.72); [5.70, 7.75]	5.65 (3.44, 8.38); [5.05, 6.46]	5.65 (3.44, 8.38); [5.05, 6.46]	6.02 (3.74, 10.60); [5.28, 6.89]
		6.44 (3.74, 10.60); [5.51, 7.33]	6.18 (3.44, 8.88); [5.30, 7.04]	5.65 (3.44, 8.38); [5.05, 6.46]	6.02 (3.74, 10.60); [5.28, 6.89]	6.94 (4.68, 8.88); [6.11, 7.49]	6.02 (3.74, 10.60); [5.28, 6.89]	6.94 (4.68, 8.88); [6.11, 7.49]	6.94 (4.68, 8.88); [6.11, 7.49]
	*p*-value	0.4829	0.6263	0.0484*	0.2977	0.7515	0.3395	0.0177*	0.1770
PLT × 10^9^/L	median (range); [IQR]	266.50 (172.00, 367.00); [235.00, 293.50]	247.50 (135.00, 415.00); [209.50, 290.00]	273.00 (172.00, 367.00); [242.00, 300.00]	273.00 (172.00, 367.00); [242.00, 300.00]	273.00 (172.00, 367.00); [242.00, 300.00]	248.00 (188.00, 355.00); [221.75, 289.00]	248.00 (188.00, 355.00); [221.75, 289.00]	229.00 (135.00, 415.00); [192.50, 257.50]
		247.50 (135.00, 415.00); [209.50, 290.00]	253.00 (171.00, 392.00); [221.50, 296.50]	248.00 (188.00, 355.00); [221.75, 289.00]	229.00 (135.00, 415.00); [192.50, 257.50]	265.00 (171.00, 392.00); [218.50, 316.00]	229.00 (135.00, 415.00); [192.50, 257.50]	265.00 (171.00, 392.00); [218.50, 316.00]	265.00 (171.00, 392.00); [218.50, 316.00]
	*p*-value	0.1410	0.6181	0.1973	0.0155*	0.8026	0.1566	0.5936	0.1211
NEU × 10^9^/L	median (range); [IQR]	3.73 (1.29, 6.72); [3.10, 4.19]	3.85 (1.78, 6.72); [3.05, 4.68]	4.10 (2.40, 6.72); [3.56, 4.90]	4.10 (2.40, 6.72); [3.56, 4.90]	4.10 (2.40, 6.72); [3.56, 4.90]	3.11 (1.29, 5.29); [2.87, 3.53]	3.11 (1.29, 5.29); [2.87, 3.53]	3.15 (1.78, 6.09); [2.61, 3.94]
		3.85 (1.78, 6.72); [3.05, 4.68]	3.50 (1.29, 5.29); [3.00, 3.97]	3.11 (1.29, 5.29); [2.87, 3.53]	3.15 (1.78, 6.09); [2.61, 3.94]	3.78 (1.99, 4.88); [3.50, 4.25]	3.15 (1.78, 6.09); [2.61, 3.94]	3.78 (1.99, 4.88); [3.50, 4.25]	3.78 (1.99, 4.88); [3.50, 4.25]
	*p*-value	0.5384	0.1768	0.0017**	0.0248*	0.2301	0.6444	0.0438*	0.2787
LYM × 10^9^/L	median (range); [IQR]	1.88 (0.95, 3.13); [1.51, 2.16]	2.01 (0.95, 3.48); [1.76, 2.27]	1.85 (0.95, 3.13); [1.49, 2.07]	1.85 (0.95, 3.13); [1.49, 2.07]	1.85 (0.95, 3.13); [1.49, 2.07]	2.01 (1.21, 2.78); [1.56, 2.17]	2.01 (1.21, 2.78); [1.56, 2.17]	2.14 (1.24, 3.48); [1.88, 2.32]
		2.01 (0.95, 3.48); [1.76, 2.27]	2.07 (1.21, 3.60); [1.69, 2.35]	2.01 (1.21, 2.78); [1.56, 2.17]	2.14 (1.24, 3.48); [1.88, 2.32]	2.25 (1.68, 3.60); [2.04, 2.49]	2.14 (1.24, 3.48); [1.88, 2.32]	2.25 (1.68, 3.60); [2.04, 2.49]	2.25 (1.68, 3.60); [2.04, 2.49]
	*p*-value	0.0038**	0.4723	0.7382	0.0639	0.0124*	0.1305	0.0168*	0.4237
MON × 10^9^/L	median (range); [IQR]	0.49 (0.21, 0.77); [0.41, 0.61]	0.49 (0.32, 0.88); [0.41, 0.59]	0.48 (0.32, 0.77); [0.39, 0.59]	0.48 (0.32, 0.77); [0.39, 0.59]	0.48 (0.32, 0.77); [0.39, 0.59]	0.52 (0.21, 0.67); [0.43, 0.62]	0.52 (0.21, 0.67); [0.43, 0.62]	0.54 (0.32, 0.88); [0.42, 0.59]
		0.49 (0.32, 0.88); [0.41, 0.59]	0.53 (0.21, 0.72); [0.44, 0.62]	0.52 (0.21, 0.67); [0.43, 0.62]	0.54 (0.32, 0.88); [0.42, 0.59]	0.54 (0.32, 0.72); [0.51, 0.62]	0.54 (0.32, 0.88); [0.42, 0.59]	0.54 (0.32, 0.72); [0.51, 0.62]	0.54 (0.32, 0.72); [0.51, 0.62]
	*p*-value	0.2250	0.4171	0.5453	0.3942	0.1471	0.7867	0.3845	0.5938
EO × 10^9^/L	median (range); [IQR]	0.12 (0.01, 0.36); [0.09, 0.20]	0.12 (0.01, 0.36); [0.07, 0.18]	0.10 (0.01, 0.36); [0.06, 0.14]	0.10 (0.01, 0.36); [0.06, 0.14]	0.10 (0.01, 0.36); [0.06, 0.14]	0.16 (0.04, 0.33); [0.12, 0.22]	0.16 (0.04, 0.33); [0.12, 0.22]	0.16 (0.03, 0.27); [0.09, 0.20]
		0.12 (0.01, 0.36); [0.07, 0.18]	0.16 (0.00, 0.57); [0.11, 0.23]	0.16 (0.04, 0.33); [0.12, 0.22]	0.16 (0.03, 0.27); [0.09, 0.20]	0.16 (0.00, 0.57); [0.10, 0.23]	0.16 (0.03, 0.27); [0.09, 0.20]	0.16 (0.00, 0.57); [0.10, 0.23]	0.16 (0.00, 0.57); [0.10, 0.23]
	*p*-value	0.4274	0.0521	0.0219*	0.1719	0.1024	0.4078	0.8588	0.4944
BAS × 10^9^/L	median (range); [IQR]	0.03 (0.01, 0.09); [0.02, 0.04]	0.03 (0.01, 0.09); (0.02, 0.04]	0.04 (0.01, 0.09); [0.03, 0.04]	0.04 (0.01, 0.09); [0.03, 0.04]	0.04 (0.01, 0.09); [0.03, 0.04]	0.03 (0.01, 0.09); [0.02, 0.04]	0.03 (0.01, 0.09); [0.02, 0.04]	0.02 (0.01, 0.08); [0.02, 0.03]
		0.03 (0.01, 0.09); [0.02, 0.04]	0.04 (0.01, 0.09); [0.02, 0.05]	0.03 (0.01, 0.09); [0.02, 0.04]	0.02 (0.01, 0.08); [0.02, 0.03]	0.04 (0.01, 0.07); [0.03, 0.06]	0.02 (0.01, 0.08); [0.02, 0.03]	0.04 (0.01, 0.07); [0.03, 0.06]	0.04 (0.01, 0.07); [0.03, 0.06]
	*p*-value	0.3311	0.1189	0.6790	0.0231*	0.3953	0.0978	0.3041	0.0196*
NLR	median (range); [IQR]	2.01 (0.76, 4.71); [1.67, 2.44]	1.92 (0.86, 4.71); [1.56, 2.39]	2.12 (1.32, 4.71); [1.87, 2.64]	2.12 (1.32, 4.71); [1.87, 2.64]	2.12 (1.32, 4.71); [1.87, 2.64]	1.76 (0.76, 2.76); [1.39, 2.05]	1.76 (0.76, 2.76); [1.39, 2.05]	1.63 (0.86, 2.62); [1.34, 2.02]
		1.92 (0.86, 4.71); [1.56, 2.39]	1.69 (0.76, 2.76); [1.27, 2.05]	1.76 (0.76, 2.76); [1.39, 2.05]	1.63 (0.86, 2.62); [1.34, 2.02]	1.51 (0.96, 2.43); [1.27, 2.02]	1.63 (0.86, 2.62); [1.34, 2.02]	1.51 (0.96, 2.43); [1.27, 2.02]	1.51 (0.96, 2.43); [1.27, 2.02]
	*p*-value	0.0157*	0.0687	0.0142*	0.0041**	0.0046**	0.7745	0.6926	0.8939
PLR	median (range); [IQR]	144.08 (90.20, 308.42); [118.44, 167.08]	123.89 (77.63, 308.42); [100.26, 160.09]	148.55 (90.20, 308.42); [121.71, 173.38]	148.55 (90.20, 308.42); [121.71, 173.38]	148.55 (90.20, 308.42); [121.71, 173.38]	137.56 (101.83, 182.64); [118.44, 152.32]	137.56 (101.83, 182.64); [118.44, 152.32]	103.61 (77.63, 187.78); [95.69, 122.58]
		123.89 (77.63, 308.42); [100.26, 160.09]	119.75 (67.52, 194.05); [107.16, 149.93]	137.56 (101.83, 182.64); [118.44, 152.32]	103.61 (77.63, 187.78); [95.69, 122.58]	116.74 (67.52, 194.05); [99.43, 134.44]	103.61 (77.63, 187.78); [95.69, 122.58]	116.74 (67.52, 194.05); [99.43, 134.44]	116.74 (67.52, 194.05); [99.43, 134.44]
	*p*-value	0.0006***	0.7719	0.4448	0.0058**	0.0388*	0.0042**	0.0753	0.4839
B) *Vaxzevria* vaccine									
IgG [BAU/mL]	median (range); [IQR]	255.40 (105.00, 449.90); [203.50, 395.20]	254.90 (105.00, 432.00); [196.40, 353.70]	215.70 (105.00, 397.80); [183.40, 303.00]	215.70 (105.00, 397.80); [183.40, 303.00]	215.70 (105.00, 397.80); [183.40, 303.00]	374.55 (178.60, 449.90); [241.23, 419.68]	374.55 (178.60, 449.90); [241.23, 419.68]	296.30 (149.10, 432.00); [234.85, 361.00]
		142.10 (85.90, 432.00); [120.28, 288.15]	178.60 (85.90, 449.90); [120.75, 374.55]	374.55 (178.60, 449.90); [241.23, 419.68]	296.30 (149.10, 432.00); [234.85, 361.00]	119.80 (85.90, 135.10); [109.50, 133.55]	296.30 (149.10, 432.00); [234.85, 361.00]	119.80 (85.90, 135.10); [109.50, 133.55]	119.80 (85.90, 135.10); [109.50, 133.55]
	*p*-value	0.0071**	0.1846	0.0062**	0.0929	0.0007***	0.2357	<0.0001***	<0.0001***
IFN-γ [mIU/mL]	median (range); [IQR]	4260.10 (1604.70, 9541.62); [3400.00, 4912.19]	4530.74 (1604.70, 12408.56); [3559.46, 5007.81]	4263.45 (1604.70, 5074.44); [3329.92, 4768.88]	4263.45 (1604.70, 5074.44); [3329.92, 4768.88]	4263.45 (1604.70, 5074.44); [3329.92, 4768.88]	4238.15 (2651.20, 9541.62); [3765.54, 6588.69]	4238.15 (2651.20, 9541.62); [3765.54, 6588.69]	4985.60 (3521.46, 12408.56); [4153.40, 7856.88]
		5362.95 (3521.46, 14110.44); [4372.26, 9423.87]	4936.90 (2651.20, 14110.44); [3912.39, 8515.200]	4238.15 (2651.20, 9541.62); [3765.54, 6588.69]	4985.60 (3521.46, 12408.56); [4153.40, 7856.88]	6909.82 (3779.15, 14110.44); [4544.67, 10105.15]	4985.60 (3521.46, 12408.56); [4153.40, 7856.88]	6909.82 (3779.15, 14110.44); [4544.67, 10105.15]	6909.82 (3779.15, 14110.44); [4544.67, 10105.15]
	*p*-value	0.0012**	0.1139	0.1826	0.0149*	0.0016**	0.2059	0.0269*	0.2717
WBC × 10^9^/L	median (range); [IQR]	6.46 (4.12, 8.94); [5.71, 7.57]	6.04 (4.12, 10.63); [5.66, 7.06]	6.04 (4.12, 7.90); [5.71, 6.50]	6.04 (4.12, 7.90); [5.71, 6.50]	6.04 (4.12, 7.90); [5.71, 6.50]	7.31 (4.40, 8.94); [6.20, 8.10]	7.31 (4.40, 8.94); [6.20, 8.10]	6.44 (4.64, 10.63); [5.12, 7.37]
		5.95 (4.62, 10.63); [5.10, 6.67]	6.27 (4.40, 8.94); [5.29, 7.49]	7.31 (4.40, 8.94); [6.20, 8.10]	6.44 (4.64, 10.63); [5.12, 7.37]	5.75 (4.62, 7.82); [5.05, 6.25]	6.44 (4.64, 10.63); [5.12, 7.37]	5.75 (4.62, 7.82); [5.05, 6.25]	5.75 (4.62, 7.82); [5.05, 6.25]
	*p*-value	0.0781	0.9015	0.0776	0.7057	0.1358	0.2059	0.0086**	0.1776
PLT × 10^9^/L	median (range); [IQR]	234.00 (149.00, 364.00); [206.00, 275.00]	250.50 (149.00, 364.00); [213.00, 289.50]	238.00 (149.00, 364.00); [206.00, 262.00]	238.00 (149.00, 364.00); [206.00, 262.00]	238.00 (149.00, 364.00); [206.00, 262.00]	234.00 (161.00, 316.00); [214.25, 282.25]	234.00 (161.00, 316.00); [214.25, 282.25]	276.00 (183.00, 349.00); [235.50, 305.00]
		252.00 (153.00, 361.00); [207.00, 289.75]	234.00 (153.00, 361.00); [205.50, 276.00]	234.00 (161.00, 316.00); [214.25, 282.25]	276.00 (183.00, 349.00); [235.50, 305.00]	223.00 (153.00, 361.00); [196.50, 276.00]	276.00 (183.00, 349.00); [235.50, 305.00]	223.00 (153.00, 361.00); [196.50, 276.00]	223.00 (153.00, 361.00); [196.50, 276.00]
	*p*-value	0.4208	0.4133	0.6787	0.1127	0.9699	0.2059	0.5665	0.1150
NEU × 10^9^/L	median (range); [IQR]	3.75 (1.81, 6.72); [2.95, 4.56]	3.77 (1.81, 7.52); [2.96, 4.10]	3.15 (1.81, 5.32); [2.88, 3.88]	3.15 (1.81, 5.32); [2.88, 3.88]	3.15 (1.81, 5.32); [2.88, 3.88]	4.15 (1.84, 6.72); [3.00, 5.44]	4.15 (1.84, 6.72); [3.00, 5.44]	3.84 (2.35, 7.52); [3.51, 4.45]
		3.76 (2.33, 7.52); [3.16, 4.55]	3.75 (1.84, 6.72); [3.05, 4.88]	4.15 (1.84, 6.72); [3.00, 5.44]	3.84 (2.35, 7.52); [3.51, 4.45]	3.57 (2.33, 5.12); [3.08, 4.45]	3.84 (2.35, 7.52); [3.51, 4.45]	3.57 (2.33, 5.12); [3.08, 4.45]	3.57 (2.33, 5.12); [3.08, 4.45]
	*p*-value	0.8580	0.4747	0.1050	0.1801	0.5332	0.6637	0.2684	0.3952
LYM × 10^9^/L	median (range); [IQR]	2.02 (0.98, 2.93); [1.75, 2.36]	2.14 (1.29, 2.83); [1.81, 2.29]	2.13 (1.29, 2.83); [1.85, 2.36]	2.13 (1.29, 2.83); [1.85, 2.36]	2.13 (1.29, 2.83); [1.85, 2.36]	1.90 (0.98, 2.93); [1.74, 2.45]	1.90 (0.98, 2.93); [1.74, 2.45]	2.14 (1.56, 2.43); [1.79, 2.22]
		1.98 (1.07, 2.85); [1.74, 2.20]	3.75 (1.84, 6.72); [3.05, 4.88]	1.90 (0.98, 2.93); [1.74, 2.45]	2.14 (1.56, 2.43); [1.79, 2.22]	1.90 (1.07, 2.85); [1.56, 2.11]	2.14 (1.56, 2.43); [1.79, 2.22]	1.90 (1.07, 2.85); [1.56, 2.11]	1.90 (1.07, 2.85); [1.56, 2.11]
	*p*-value	0.4051	0.2426	0.6525	0.6504	0.1801	0.8125	0.4526	0.1985
MON × 10^9^/L	median (range); [IQR]	0.51 (0.24, 0.85); [0.47, 0.62]	0.53 (0.24, 0.82); [0.48, 0.59]	0.51 (0.24, 0.76); [0.44, 0.58]	0.51 (0.24, 0.76); [0.44, 0.58]	0.51 (0.24, 0.76); [0.44, 0.58]	0.52 (0.46, 0.85); [0.48, 0.63]	0.52 (0.46, 0.85); [0.48, 0.63]	0.57 (0.41, 0.82); [0.51, 0.61]
		0.53 (0.29, 0.82); [0.48, 0.59]	0.50 (0.29, 0.85); [0.47, 0.62]	0.52 (0.46, 0.85); [0.48, 0.63]	0.57 (0.41, 0.82); [0.51, 0.61]	0.50 (0.29, 0.76); [0.43, 0.55]	0.57 (0.41, 0.82); [0.51, 0.61]	0.50 (0.29, 0.76); [0.43, 0.55]	0.50 (0.29, 0.76); [0.43, 0.55]
	*p*-value	0.8688	0.6600	0.3308	0.1262	0.8502	0.6637	0.1727	0.0745
EO × 10^9^/L	median (range); [IQR]	0.17 (0.01, 0.68); [0.10, 0.24]	0.10 (0.01, 0.34); [0.07, 0.18]	0.16 (0.01, 0.34); [0.05, 0.20]	0.51 (0.24, 0.76); [0.44, 0.58]	0.51 (0.24, 0.76); [0.44, 0.58]	0.18 (0.01, 0.68); [0.15, 0.26]	0.18 (0.01, 0.68); [0.15, 0.26]	0.10 (0.04, 0.30); [0.08, 0.14]
		0.12 (0.04, 0.57); [0.08, 0.18]	0.15 (0.01, 0.68); [0.12, 0.24]	0.18 (0.01, 0.68); [0.15, 0.26]	0.10 (0.04, 0.30); [0.08, 0.14]	0.12 (0.08, 0.57); [0.10, 0.21]	0.10 (0.04, 0.30); [0.08, 0.14]	0.12 (0.08, 0.57); [0.10, 0.21]	0.12 (0.08, 0.57); [0.10, 0.21]
	*p*-value	0.1584	0.0237*	0.1171	0.6917	0.5332	0.0086**	0.0969	0.1150
BAS × 10^9^/L	median (range); [IQR]	0.03 (0.01, 0.06); [0.02, 0.04]	0.03 (0.01, 0.06); [0.02, 0.04]	0.03 (0.01, 0.06); [0.02, 0.04]	0.03 (0.01, 0.06); [0.02, 0.04]	0.03 (0.01, 0.06); [0.02, 0.04]	0.04 (0.02, 0.06); [0.03, 0.05]	0.04 (0.02, 0.06); [0.03, 0.05]	0.03 (0.01, 0.05); [0.03, 0.05]
		0.03 (0.01, 0.07); [0.02, 0.04]	0.03 (0.02, 0.07); [0.02, 0.04]	0.04 (0.02, 0.06); [0.03, 0.05]	0.03 (0.01, 0.05); [0.03, 0.05]	0.03 (0.02, 0.07); [0.02, 0.04]	0.03 (0.01, 0.05); [0.03, 0.05]	0.03 (0.02, 0.07); [0.02, 0.04]	0.03 (0.02, 0.07); [0.02, 0.04]
	*p*-value	0.5222	0.4620	0.1171	0.5840	0.9849	0.3738	0.1232	0.5897
NLR	median (range); [IQR]	1.79 (0.78, 4.38); [1.31, 2.65]	1.77 (1.06, 3.51); [1.34, 2.37]	1.38 (1.06, 2.97); [1.28, 2.05]	1.38 (1.06, 2.97); [1.28, 2.05]	1.38 (1.06, 2.97); [1.28, 2.05]	2.11 (0.78, 4.38); [1.56, 2.98]	2.11 (0.78, 4.38); [1.56, 2.98]	1.91 (1.11, 3.51); [1.56, 2.38]
		1.96 (1.11, 3.82); [1.66, 2.36]	2.04 (0.78, 4.38); [1.65, 2.62]	2.11 (0.78, 4.38); [1.56, 2.98]	1.91 (1.11, 3.51); [1.56, 2.38]	1.98 (1.18, 3.82); [1.82, 2.28]	1.91 (1.11, 3.51); [1.56, 2.38]	1.98 (1.18, 3.82); [1.82, 2.28]	1.98 (1.18, 3.82); [1.82, 2.28]
	*p*-value	0.4090	0.1489	0.1303	0.1358	0.1262	0.6073	0.7820	0.6936
PLR	median (range); [IQR]	115.68 (62.08, 223.31); [95.34, 135.38]	114.85 (62.08, 223.72); [96.66, 153.24]	107.56 (62.08, 223.31); [93.72, 121.89]	107.56 (62.08, 223.31); [93.72, 121.89]	107.56 (62.08, 223.31); [93.72, 121.89]	130.46 (68.51, 221.43); [109.04, 135.71]	130.46 (68.51, 221.43); [109.04, 135.71]	1.91 (1.11, 3.51); [1.56, 2.38]
		131.06 (62.46, 223.72); [107.30, 159.08]	128.37 (62.46, 221.43); [107.55, 144.73]	130.46 (68.51, 221.43); [109.04, 135.71]	1.91 (1.11, 3.51); [1.56, 2.38]	1.98 (1.18, 3.82); [1.82, 2.28]	1.91 (1.11, 3.51); [1.56, 2.38]	1.98 (1.18, 3.82); [1.82, 2.28]	1.98 (1.18, 3.82); [1.82, 2.28]
	*p*-value	0.1958	0.4747	0.2641	0.1681	0.1681	0.5532	0.8125	0.9835

*p ≤ 0.05, **p ≤ 0.01, ***p ≤ 0.001 statistical significance.

For the Comirnaty vaccine, the following parameters changed significantly:

- WBC: Between healthy individuals who had received the second or third dose and those who had recovered from COVID-19 and had received the third dose (*p* ≤ 0.05).
- NEU: Between healthy individuals who had received the second or third dose (*p* ≤ 0.01) and those who had recovered from COVID-19 and had received the second or third dose (*p* ≤ 0.05).
- LYM: Between both healthy individuals and those who had recovered from COVID-19 (*p* ≤ 0.01) and between healthy individuals vaccinated with the second or third dose and those who had recovered after receiving the same number of doses (*p* ≤ 0.05).
- EO: In healthy individuals who had received the second or third dose (*p* ≤ 0.05).
- BAS: Between healthy individuals and those who had recovered from COVID-19 after receiving the second dose and those who had recovered after being vaccinated with the second or third dose (*p* ≤ 0.05).
- NLR: Between both healthy individuals and those who had recovered from COVID-19 (*p* ≤ 0.05), between healthy individuals who had received second or third dose (*p* ≤ 0.05), and between both healthy and recovered individuals who had been vaccinated twice (*p* ≤ 0.01) or thrice (*p* ≤ 0.01).
- PLR: Between healthy individuals and those who had recovered from COVID-19 (*p* ≤ 0.001) and between healthy and recovered individuals vaccinated twice (*p* ≤ 0.05) or thrice (*p* ≤ 0.01).

No statistically significant differences were observed for the PLT and MON parameters after the Comirnaty vaccination (**Table 2A**).

For the Vaxzevria vaccine, statistically significant changes were observed only in the following parameters:

- WBC: Between healthy individuals and those who had recovered from COVID-19 having received the third dose (*p* ≤ 0.01).
- EO: Between healthy individuals who had received the second or third dose (*p* ≤ 0.05) and between healthy individuals who had been vaccinated thrice and those who had recovered after being vaccinated twice (*p* ≤ 0.01).

No statistically significant differences were observed for the PLT, NEU, LYM, MON, BAS, NLR, or PLR parameters after Vaxzevria vaccination (**Table 2B**).

## Discussion

The primary objective of this study was to compare the adaptive immune responses elicited by the Comirnaty (Pfizer) and Vaxzevria (AstraZeneca) vaccines in healthy individuals, both with and without prior SARS-CoV-2 infection. Immune responses were assessed based on anti-SARS-CoV-2 Immunoglobulin G (IgG) antibody and specific interferon-gamma (IFN-γ) release assay (IGRA) cytokine levels, as well as leukocyte profile markers, including neutrophil-to-lymphocyte ratio (NLR) and platelet-to-lymphocyte ratio (PLR). This comprehensive approach enabled the evaluation of both humoral and cellular immunity across different vaccination regimens and infection histories.

Our findings demonstrated that individuals vaccinated with Comirnaty exhibited significantly higher levels of IgG antibodies against the S protein compared to those who received Vaxzevria. These differences were particularly evident after the third dose. Similarly, IFN-γ production was significantly higher in individuals who received Comirnaty vaccination, suggesting a stronger T-cell-mediated immune response. In the context of cellular response, a significantly higher number of IFN-γ-secreting.

T lymphocytes was detected in individuals vaccinated with Comirnaty compared to Vaxzevria recipients. Despite a higher percentage of total cytotoxic lymphocytes in Vaxzevria recipients, the lower number of IFN-γ-producing T lymphocytes, alongside a reduced percentage of CD4+ lymphocytes, may suggest a diminished effector function of this segment of the immune response following Vaxzevria administration (10).

These observed differences can be attributed to the distinct mechanisms of action of the two vaccine platforms production (8, 9). Comirnaty, an mRNA vaccine, utilizes lipid nanoparticles encapsulating mRNA directed against the S1 protein (1, 2). Upon degradation of these lipid nanoparticles in endosomes, mRNA molecules are released into the cytoplasm of myocytes, initiating S protein production (3–5, 11, 12). The synthesized S protein or its fragments are then presented on the cell surface and secreted into the interstitial fluid and blood, facilitating rapid phagocytosis by antigen-presenting cells (APCs) and subsequent presentation to Th and B lymphocytes (4, 11–13). Furthermore, the inherent adjuvant properties of mRNA, associated with the cellular reaction to foreign RNA in the cytosol, support a more intensive immune response (11, 12, 14–18). Studies have also indicated a greater intensity of plasmablast formation after mRNA vaccine administration compared to adenoviral vector vaccines (4, 10, 12, 13). These results underscore the clinical relevance of selecting mRNA vaccines, such as Comirnaty, particularly in SARS-CoV-2 naïve individuals, who may benefit from their superior ability to induce robust humoral and cellular immune responses. This finding may inform public health policies, especially in the context of primary vaccination strategies and booster planning.

In contrast to Comirnaty’s mRNA, which is directly introduced into the cytoplasm and protected by lipid nanoparticles, the DNA-based platform used by Vaxzevria may offer less protection against degradation and may induce a lower magnitude of immune response. Moreover, residual host cell proteins or adenoviral components present in the final vaccine formulation could attenuate the strength of the humoral response, potentially contributing to the observed differences in antibody titers between vaccine recipients (18, 19).

A distinct immune response pattern was observed in individuals who had previously contracted COVID-19 and were subsequently vaccinated (20). Irrespective of vaccine type, participants with a history of COVID-19 infection displayed significantly higher immune responses (IgG and IFN-γ levels) compared to those without prior exposure, suggesting that natural infection contributes to enhanced immunological memory upon vaccination. While hybrid immunity (following natural infection and vaccination) is generally considered more robust and durable than natural immunity or vaccine-acquired immunity alone (21), although the present study initially suggested no significant differences between vaccinated-only and hybrid immunity groups, the quantitative data indicate that hybrid immunity is associated with significantly elevated IgG and IFN-γ levels. This discrepancy may reflect differences in statistical power within subgroups or the timing of sample collection, and highlights the complex interplay between natural and vaccine-induced immunity. This highlights the complex interplay between natural and vaccine-induced immunity, warranting further investigation.

Interestingly, in the Vaxzevria-vaccinated group with a prior infection history, a decrease in antibody concentration was observed after the third dose (20). This phenomenon could be associated with immune exhaustion, where repeated antigen exposure leads to impaired function of effector cells (22). An alternative explanation may lie in the differing dynamics of immune responses based on the vaccine platform, with vector-based vaccines potentially eliciting a shorter duration of immune response compared to mRNA-based vaccines (23, 24).

The administration of a third vaccine dose significantly boosted both IgG and IFN-γ levels, thereby emphasizing the critical importance of booster doses in maintaining robust and long-term immunity against SARS-CoV-2, especially in individuals who had no prior infection. For the Comirnaty vaccine, subsequent doses consistently increased IgG and IFN-γ concentrations. However, in the Vaxzevria-vaccinated groups, a significant increase in IFN-γ concentrations was not observed after subsequent doses (21, 25), which might be attributed to the insufficient sample size in those cohorts.

Further hematological analysis revealed notable differences in the immune activation patterns between the vaccine types, specifically concerning white blood cell counts (WBC), neutrophil-to-lymphocyte ratio (NLR), and platelet-to-lymphocyte ratio (PLR). NLR and PLR are recognized inflammatory markers that reflect systemic immune activation (26, 27). The observed differences in these ratios between vaccine platforms may suggest distinct activation of immune pathways (28–30).

It is important to acknowledge that various SARS-CoV-2 variants, such as Alpha (B.1.1.7) and Delta (B.1.617.2) and its sublineages (AY.4, AY.122), circulated in Poland during the study period (31). These variants were characterized by increased transmissibility and partial immune escape. Specifically, mutations in the spike protein, particularly within the receptor-binding domain (RBD), could lead to reduced neutralization by vaccine-induced antibodies (32). These factors may have influenced the observed post-vaccination immune responses.

Another limitation of this study is the gender imbalance within the cohort (93 women vs. 41 men) and the absence of an analysis of sex-based differences (22). Women typically exhibit stronger humoral and cellular immune responses compared to men, influenced partly by sex hormones and variations in immune system-related gene expression on the X chromosome (33, 34). In the context of SARS-CoV-2 vaccination, women have shown higher antibody titers and more intense cytokine production (e.g., IFN-γ), particularly after mRNA-based vaccines, and they also more frequently report adverse events, possibly due to a more robust immune activation (35). These considerations are crucial for interpreting immunological study outcomes and for designing effective vaccination strategies. The gender imbalance may limit the generalizability of the results, as sex-related immune differences could have skewed overall response profiles. Future studies should incorporate stratified analyses by sex to better understand immunogenicity patterns.

Additionally, the lack of variant-specific immune response analysis is a limitation, particularly considering the circulation of immune-evasive strains like Delta and Alpha during the study period. Variants may differentially impact vaccine effectiveness, and their inclusion in future studies is essential to ensure the robustness of immunization strategies.

Despite these limitations, this study stands as one of the few in the scientific literature comparing the Comirnaty and Vaxzevria vaccines in a Central European population. The findings contribute to a better understanding of vaccine-induced immunity and support the ongoing optimization of COVID-19 vaccination strategies globally. Future research should focus on assessing long-term immunity, vaccine-induced protection against emerging SARS-CoV-2 variants, and the potential need for additional booster doses in diverse population groups.

## Conclusion

- Differential Immune Responses: This study demonstrated that Comirnaty (Pfizer) induces a stronger humoral and cellular immune response than Vaxzevria (AstraZeneca), as evidenced by higher IgG concentrations and increased IFN-γ production, particularly after the third dose.
- Effect of Prior SARS-CoV-2 Infection: Regardless of vaccine type, participants with a history of COVID-19 infection exhibited significantly higher immune responses than those without prior exposure, suggesting that natural infection contributes to enhanced immunological memory upon vaccination.
- Importance of Booster Doses: The third dose significantly enhanced both IgG and IFN-γ levels, underscoring the necessity of booster doses to maintain long-term immunity against SARS-CoV-2, particularly in individuals who had not been previously infected.
- Variations in Hematologic Parameters: Differences in WBC counts, NLR, and PLR between vaccine groups indicated distinct immune activation patterns, potentially influencing vaccine effectiveness and long-term immunity.
- Implications for Vaccine Strategy: Given the observed differences in immune responses, mRNA-based vaccines, such as Comirnaty, may provide more robust protection against SARS-CoV-2, especially in individuals who had not been infected previously. However, an adenoviral vector vaccine, such as Vaxzevria, still plays a crucial role in broadening population immunity.
- Future Considerations: Further research should focus on long-term immunity, vaccine-induced protection against emerging SARS-CoV-2 variants and the potential need for additional booster doses to sustain immunity in different population groups.

These findings contribute to a better understanding of vaccine-induced immunity and support the ongoing optimization of COVID-19 vaccination strategies worldwide.

### Study strengths and limitations

This is one of the few studies in the scientific literature comparing the Comirnaty and Vaxzevria vaccines in a Central European population, serving as a starting point for further discussion on their biology, pharmacology, indications, contraindications, and adverse effects, with the aim of better understanding these two vaccine preparations.

## Data Availability

The original contributions presented in the study are included in the article/supplementary material. Further inquiries can be directed to the corresponding author.
